# Are Palliative Interventions Worth the Risk in Advanced Gastric Cancer? A Systematic Review

**DOI:** 10.3390/jcm13195809

**Published:** 2024-09-28

**Authors:** Alicia A. Gingrich, Renceh B. Flojo, Allyson Walsh, Jennifer Olson, Danielle Hanson, Sarah B. Bateni, Sepideh Gholami, Amanda R. Kirane

**Affiliations:** 1 Department of Surgery, MD Anderson Cancer Center, Houston, TX 77030, USA; alicia.ag.12@gmail.com; 2 Department of Surgery, Section of Surgical Oncology, Stanford University, 1201 Welch Road MSLS 214, Palo Alto, CA 94305, USA; rflojo@stanford.edu; 3 Department of Surgery, UC Davis, Sacramento, CA 95817, USA; alwalsh@ucsd.edu (A.W.); hanson83@msu.edu (D.H.); 4 Fox Chase Cancer Center, Philadelphia, PA 19111, USA; jlols@ucdavis.edu; 5 Department of Surgery, Northwell Health, New Hyde Park, NY 11040, USA; sbbateni@uabmc.edu; 6 Department of Surgery, University of Alabama Birmingham, Birmingham, AL 35294, USA; sgholami@northwell.edu

**Keywords:** gastric cancer, palliative surgery, chemotherapy

## Abstract

**Background:** Less than 25% of gastric cancers (GC) are discovered early, leading to limited treatment options and poor outcomes (27.8% mortality, 3.7% 5-year survival). Screening programs have improved cure rates, yet post-diagnosis treatment guidelines remain unclear (systemic chemotherapy versus surgery). The optimal type of palliative surgery (palliative gastrectomy (PG), surgical bypass (SB), endoscopic stenting (ES)) for long-term outcomes is also debated. **Methods:** A literature review was conducted using PubMed, MEDLINE, and EMBASE databases along with Google Scholar with the search terms “gastric cancer” and “palliative surgery” for studies post-1985. From the initial 1018 articles, multiple screenings narrowed it to 92 articles meeting criteria such as “metastatic, stage IV GC”, and intervention (surgery or chemotherapy). Data regarding survival and other long-term outcomes were recorded. **Results:** Overall, there was significant variation between studies but there were similarities of the conclusions reached. ES provided quick symptom relief, while PG showed improved overall survival (OS) only with adjuvant chemotherapy in a selective population. PG had higher mortality rates compared to SB, with ES having a reported 0% mortality, but OS improved with chemotherapy across both SB and PG. **Conclusions:** Less frail patients may experience an improvement in OS with palliative resection under limited circumstances. However, operative intervention without systemic chemotherapy is unlikely to demonstrate a survival benefit. Further research is needed to explore any correlations.

## 1. Introduction

In the United States and Europe, less than 25% of gastric cancer (GC) is discovered in the early stages, leading to lack of curative interventions and a dismal prognosis [1]. As a result of late presentation, it is associated with high mortality, with 27.8% and 3.7% 5-year survival in late-stage regional and distant disease, respectively [1,2]. While its late presentation can prove challenging, in Asian countries such as Japan and Korea, where screening programs are established, over 50% of GC is discovered in the early stages, which corresponds to a greater than 90% cure rate [3,4,5]. But sadly, guidelines for GC screening have not been standardized between countries, leading to its variable cure rates. 

In the event it is not caught, the traditional approach for treatment is palliative surgery in gastric cancer only when a patient becomes symptomatic. However, a recent study of the National Inpatient Sample (NIS) by Solsky examined the outcomes of patients undergoing emergent resections [6]. These patients represented 12% of the overall GC population and were statistically more likely to present with bleeding, perforation, or obstruction. Such presentations were associated with worse outcomes including doubling in-hospital mortality, prolonged length of hospital stay, and almost 40% of patients being discharged to a facility and/or dying in the hospital [6]. Considering this, we conducted a systematic review to determine the role of elective surgical intervention with palliative intent in the management of late-stage GC. We also sought to determine how non-operative interventions compared with operative interventions to control symptomatic, late-stage GC. 

## 2. Methods

### 2.1. Data Sources

Utilizing the assistance of the UC Davis Blaisdell Medical Library, an electronic literature search was conducted in the PubMed, MEDLINE, and EMBASE databases, as well as on Google Scholar. Search terms included “gastric cancer” and “palliative surgery”. Searches were limited to the English language and studies occurring after 1985. The search was not limited by geographic location, and articles from all countries were considered so long as they were translated into English. Any unavailable full text articles were requested through the UC Davis library resources. If the library was unable to locate the full text, no additional attempts were made. We did not attempt to locate unpublished material. 

### 2.2. Review Process

**Figure 1:**
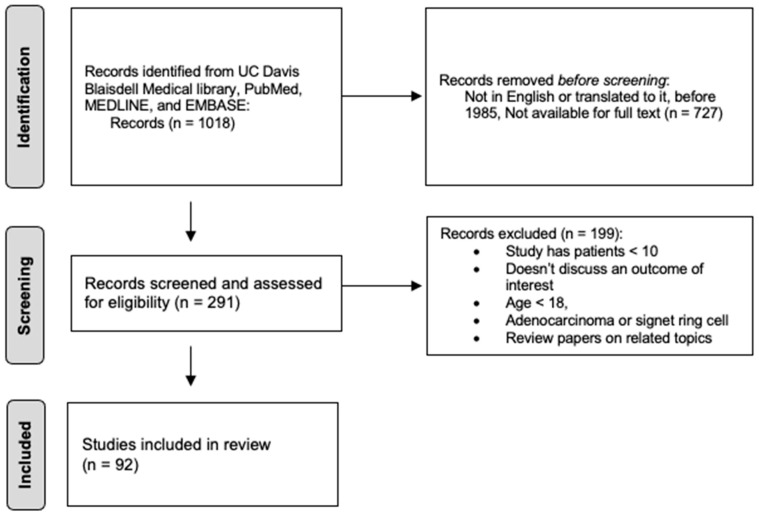
PRISMA Flow diagram of the selection of reviewed articles.

The electronic literature search returned 1018 articles for consideration. All titles and abstracts were screened for relevance. Following screening, 291 full-text articles were assessed for eligibility. To qualify, a study had to have a population with metastatic, Stage IV gastric cancer. An intervention with either palliative gastrectomy (PG), surgical bypass (SB), or endoscopic stenting (ES) was required. Patients were considered a control group if they did not undergo palliative intervention. Not all studies had a control group as some were case series. All studies were required to have greater than 10 patients. The study had to also discuss an outcome of interest, which was symptom palliation, post-operative complications, 30-day mortality, compliance with chemotherapy, and/or survival. Exclusion criteria were studies on patients < 18 years old, in which the primary disease was not GC (i.e., pancreatic cancer with gastric outlet obstruction), no adenocarcinoma or signet ring cell (i.e., GIST, carcinoid), and any study with less than 10 patients. Complications for surgical procedures tended to include those under Clavien-Dindo classifications. Complications for stenting procedures had much more inclusive criteria, including symptoms such as abdominal pain not requiring intervention, which were not captured in surgical studies. Previous review papers on related topics were not included (Figure 1). PRISMA guidelines were followed when conducting this study. Titles, abstracts, and full texts were reviewed separately by two reviewers and independently recorded on a spreadsheet. Discrepancies were resolved at a consensus meeting. 

### 2.3. Data Extraction

In total, 92 articles were selected for data extraction and qualitative synthesis. Data were extracted from all articles meeting inclusion criteria and with study designs pertaining to outcomes of interest. Articles were classified by topic as (1) PG compared to no operation/diagnostic laparoscopy, (2) PG compared to SB, (3) PG compared to ES, (4) SB compared to ES, (5) SB alone, or (6) ES alone. Data on patient outcomes were then abstracted based on all available data from each article, although most articles did not include all outcomes of interest. Data regarding survival, multivariate analyses, morbidity, mortality, post-intervention complications, return to oral intake, length of stay, clinical success, and completion of chemotherapy were recorded. Given the heterogeneity of study designs and available data, a meta-analysis was not conducted. 

## 3. Results

Of the articles meeting inclusion criteria, 54 discussed PG compared to no operation/diagnostic laparoscopy. From this group, 4 were prospective cohort studies and 50 were retrospective reviews. Twelve articles compared PG to SB, one of which was a prospective study. No articles have directly compared PG to ES. Eight articles compared SB to ES, including one prospective study and one randomized trial. Seven retrospective studies examined SB alone and seven examined ES alone, two of which were prospective. One article from Sweden by Keränen et al. compared all three methods among 97 patients in a retrospective study [7]. The authors of this study concluded that in patients clinically suitable for surgery, PG was associated with survival benefit. In patients who were not surgical candidates—as defined by poor performance status, high age, and extent of metastatic disease—ES provided fast, efficient relief of symptoms [7]. A summary of article types and level of evidence is shown in Table 1. 

Among the articles studying PG when compared to no operation or diagnostic laparoscopy, 24/54 articles found an association with PG to improved overall survival (OS) (*p* ≤ 0.05) among all comers, whereas 10 articles did not demonstrate an association (*p* ≥ 0.05). However, on multivariate analyses, 26/54 of the studies demonstrated an association with OS when controlling for multiple variables, which resulted in the reversal of the original univariate analysis in some studies. Of the additional variables, 10 studies found an association of PG with improved OS, but only if the patients were able to complete adjuvant chemotherapy [42,43,44,45,46,47,48,49,50,51]. Multiple other studies demonstrated an association of PG with improved OS in the setting of reduced disease burden and a younger patient population with favorable nutrition metrics [52]. Survival benefits were associated with completion of adjuvant chemotherapy, singular site of metastases (vs. multiple), absence or fewer peritoneal, lymph node or hepatic metastases, age < 60, and nutrition metrics (albumin > 3, weight loss < 5%) across more than one study. All variables associated with improved OS are summarized in Table 2. 

Of the articles returned in our review, 12 directly compared SB (gastrojejunostomy) to PG in terms of survival and procedure-associated mortality. Significant variation was seen in the results between studies. Among the articles comparing PG to SB, 4 of the 12 found an association with improved OS with PG, *p* < 0.001, including the only prospective study in this group. Three studies found no association between PG and improved OS when compared to SB. One study found that PG with an R0 resection correlated with improved OS, whereas another which demonstrated distal but not total PG was associated with survival benefit. Given the findings from Table 2, in which chemotherapy with surgery was repeatedly associated with improved survival, one study demonstrated that SB with chemotherapy had a longer median survival (354 days) when compared to PG alone (247 days) and was statistically significant (*p* = 0.0005). Among reported mortality ranges, PG had the highest 30-day mortality (0–24.1%) compared to SB (0–6%). 

Our final objective was to compare operative vs. non-operative interventions to control symptoms in late-stage GC. Among the articles comparing SB to ES, two of the eight found an association with improved OS with SB, *p* = 0.003 and *p* < 0.001. ES has a reported mortality of 0% across all studies. Morbidity and complications were widely varied among SB vs. ES studies in terms of both frequency and definition with overall morbidity for PG 10.4–88.9%, SB 22.0–80.0%, and ES 16.7–45.9%. Length of stay ranged from 11.56–41.0, 7.0–19.0, and 0.94–12.8 days for PG, SB, and ES, respectively. Return to oral intake was not reported in any studies focused on PG but was 4.9–8.0 days for SB and 1.0–2.1 days for ES. Clinical success, defined broadly as resolution of inciting symptoms, was reported for SB as 77.3–98.6% and ES as 77.3–100% (Table 3). 

## 4. Discussion

In this systematic review, we examined interventions for late-stage GC in two ways. First, we determined the effect of elective palliative gastrectomy on OS, prior to emergent surgery for symptom management, including bleeding, perforation, and obstruction. Based on the available literature, which is predominantly retrospective in nature, an association between PG and improved survival was seen most consistently in the setting of completed adjuvant chemotherapy, lower burden of disease, and a fit patient population. Secondly, we examined the clinical success of minimally invasive interventions (SB and ES). Clinical survival outcomes were equivalent, with return to oral intake faster and length of stay shorter for ES. Of note, complications appeared equivalent between groups, but metrics to define complications were very dissimilar between studies and are comparatively over-reported in ES studies. 

Numerous single and multi-center retrospective studies have been conducted in attempts to delineate the circumstances under which to proceed with surgery in the setting of metastatic disease. Taken together, the data largely support that there exists a subset of patients who demonstrate improved OS when compared to non-resection operations. These studies have attempted to identify predictors of overall survival (OS) on multivariate analysis, including decreased age, improved function status, favorable nutrition markers, and lesser extent of disease. Completion of adjuvant chemotherapy was associated with improved OS reached in most studies, and some authors speculated that PG reduces disease burden (and in some cases a more extensive lymphandectomy may be necessary) and allows for a greater response to salvage chemotherapy [67,68]. These retrospective studies lend support to the need for further prospective trials.

Our literature review returned 1018 articles, of which 92 were ultimately selected for data extraction, and most articles were retrospective in nature. However, there exist prospective randomized controlled trials that were not returned based on our search terms and will be discussed here. The first is the multi-institutional phase III trial (The REGATTA) from Japan, Korea, and Singapore, which examined 175 patients with advanced disease with single non-curable spread of disease confined to the liver (H1), peritoneum (P1), or para-aortic lymph nodes (16a1/b2) comparing gastrectomy plus adjuvant chemotherapy to chemotherapy alone [69]. This study failed to demonstrate a survival benefit for patients undergoing gastrectomy. The authors also noted that 36% of the patients in the gastrectomy group were unable to complete chemotherapy vs. 28% in the non-surgical group due to adverse events. 

The GYMSSA trial from the United States took a more aggressive approach. Seventeen patients with peritoneal carcinomatosis of gastric origin were randomized to undergo a gastrectomy (including omentectomy and D2 lymphadenectomy), metastasectomy with hyperthermic intraperitoneal chemotherapy (HIPEC) and systemic FOLFOXIRI (GYMS arm) versus systemic chemotherapy alone (SA arm) [70]. Metastatic disease sites were restricted to the peritoneum, lung, or liver and all participants had an ECOG score less than 2. Of the nine patients in the GYMS arm, three required re-operation within 30 days due to complications and four were unable to start chemotherapy. The survival amongst the group was highly variable. 

A third prospective Phase II trial from Germany evaluated the therapeutic effects of neoadjuvant chemotherapy in prolonging survival (AIO-FLOT3 trial) [71]. The authors examined patients in three arms: resectable disease, limited metastatic disease, and extensive metastatic disease. Patients with limited metastatic disease were restaged following neoadjuvant chemotherapy. The study concluded that patients with limited metastatic disease who received neoadjuvant chemotherapy and proceeded to surgery showed favorable survival compared to those not receiving surgery and those with extensive metastatic disease, indicating that a super-selected group of patients who demonstrate greater response to neoadjuvant chemotherapy may have favorable tumor biology as well as surgeon-led discretion to proceed with resection. It is important to note that, in this study, limited metastatic disease was confined to retroperitoneal lymph nodes and liver metastases, with two patients also demonstrating adrenal and pericardial involvement. Thus, the results cannot be extrapolated to patients with peritoneal disease, and therapeutic management of patients with cytologic disease only is outside the scope of our focus. But in cases where there is peritoneal involvement, there is the possibility of using endoperitoneal chemotherapeutics [72]. AIO-FLOT4, a continuation of AIO-FLOT3 trial, further corroborated these findings and emphasized that, after neoadjuvant chemotherapy, the disease must be restaged, and once the response was determined, surgery can proceed [73]. 

In terms of non-resection palliative interventions, SB by either open gastrojejunostomy (OGJ) or laparoscopic gastrojejunostomy (LGJ) has been compared to ES most frequently in single-center retrospective studies. Clinical success rates are comparable between the two, with ES consistently associated with a shorter length of stay and faster return to oral intake. Most retrospective studies are confounded by significant differences in baseline characteristics of patients, particularly the higher ECOG scores in the ES group. One randomized controlled trial by Fiori et al. examined 18 patients undergoing either SB or ES for outcomes specific to length of procedure, morbidity and mortality rate, restoration of oral intake, and gastric emptying at 8, 15, and 90 days from treatment [74]. There were no significant differences between interventions for morbidity, mortality, delayed gastric emptying, and clinical outcomes at 90 days. ES was superior with respect to faster return to oral intake, shorter operative times, and median hospitalization [75]. Taken together with the retrospective data, ES is a safe and effective alternative to SB for palliation of late-stage GC symptoms.

Recent interest has been shown in answering this question in an era of modern effective therapy for stage IV disease, where we are seeing surgical indications expand. Work by Cowling et al. examining survival data of patients undergoing PG found that they were associated with small improvement when compared to non-resectional surgery and chemotherapy, but post-operative survival benefits are less clear and carry expense of increased complications [76]. Pinto et al. and Elameh et al. further corroborated our findings that, in late-stage GC, PG was associated with improved OS when compared to SB, and PG may improve OS when combined with systemic chemotherapy, respectively [76,77]. However, a study by Minstrini et al. did not include examination of non-operative palliative intervention compared to operative interventions as discussed in our work, demonstrating the additional important information presented in this comprehensive review [78,79]. 

In conclusion, less frail patients with good performance status and small metastatic disease burden who receive palliative resection may experience improved OS in very limited circumstances. Operation in the emergent setting is associated with significant morbidity and adequate palliation may be achieved by less invasive treatment modaities. Studies demonstrate equivalent clinical success, shorter LOS, and faster return to oral intake with ES. Additionally, the data suggest that operative intervention without associated chemotherapy is unlikely to demonstrate a survival benefit. When considering operative intervention, it is ideal if the patient is able to withstand both surgery and chemotherapy. Taken together, the current research suggests that palliative GC surgery can be a reasonable choice in highly selected patients and, as therapies evolve, the potential for clinical benefit may improve. Future prospective, randomized trials would be beneficial to determine any correlations. 

## Figures and Tables

**Table 1:** Summary of 92 articles selected for systematic review by topic and level of evidence.

Interventions	Number of Articles	Level of Evidence
PG vs. No operation/No resection	54	4 Prospective cohort studies 50 Retrospective cohort studies
PG vs. SB	12	1 Prospective cohort study 11 Retrospective cohort studies
SB vs. ES	8	1 Randomized controlled trial 1 Prospective cohort study 6 Retrospective cohort studies
PG vs. ES	0	N/A
SB alone	7	7 Retrospective cohort studies
ES alone	6	2 Prospective cohort studies 4 Retrospective cohort studies
PG vs. SB vs. ES	1	1 Retrospective cohort study

PG: palliative gastrectomy, SB: surgical bypass, ES: endoscopic stent. References included outside of the directly referenced articles but included in the articles selected: [8,9,10,11,12,13,14,15,16,17,18,19,20,21,22,23,24,25,26,27,28,29,30,31,32,33,34,35,36,37,38,39,40,41].

**Table 2:** Variables associated with improved survival in palliative gastrectomy (reported hazard ratio < 1).

Author	Year	No. of Patients	Intervention	Median Survival	*p* Value
Chang, Y.R., et al. [42]	2012	108	PG with chemotherapy	12.7 mo	0.0107
		57	Nonresection with chemotherapy	11.2 mo	
		57	PG, no chemotherapy	4.8 mo	0.151
		35	No resection, no chemotherapy	4.1 mo	
Chen, S., et al. [53]	2012	42	PG Patient with lymph node metastases	13.75 mo	<0.001
		38	No surgery Patient with lymph node metastases	8.5 mo	
		25	PG + hepatectomy, Patient with liver metastases	28.9 mo	<0.001
		29	PG without hepatectomy, Patient with liver metastases	18.5 mo	
		106	No surgery, Patient with liver metastases	13.8 mo	
		107	PG, Late-stage patient	25.1 mo	0.0107
		85	No surgery, Late-stage patient	10.6 mo	
		21	PG, Multi-organ metastases	7.9 mo	>0.05
		83	No surgery, Multi-organ metastases	7.8 mo	
Cheon, S.H., et al. [54]	2008	22	PG + hepatectomy, R0 resection	17 mo	<0.001
		19	PG + hepatectomy, R1/2 + RFA	21.7 mo	0.1963
		17	Palliative gastrectomy	8.1 mo	0.0184
Cheong, J.-H., et al. [55]	2007	154	Gastrectomy +/− cytoreductive resection + intraperitoneal chemo	11.4mo	0.018
		R0 = 37	Gastrectomy +/− cytoreductive resection + intraperitoneal chemo	25.5	<0.001
		R1 = 56	Gastrectomy +/− cytoreductive resection + intraperitoneal chemo	15.6	
		R2 = 61	Gastrectomy +/− cytoreductive resection + intraperitoneal chemo	7.2	
Chiu, C.-F., et al. [56]	2016	137	PG age < 60	16.9 mo	<0.001
			No surgery < 60	7.6 mo	
			PG age > 60	8.7 mo	0.252
			No surgery > 60	6.6 mo	
		137	PG with normal CEA/CA 19-9	14.7 mo	0.001
			No surgery with normal CEA/CA 19-9	7.1mo	
			PG with high CEA/CA 19-9	5.2 mo	0.271
			No surgery with high CEA/CA 19-9	5.5 mo	
Hartgrink, H.H., et al. [57]	2002	105	Palliative gastrectomy, Patient with 1 “positive” sign ^†^	10.5 mo	0.034
		44	No resection, Patient with 1 “positive” sign	6.7 mo	
		51	Palliative gastrectomy, Patient with 2 or more “positive” signs	5.7 mo	0.084
		85	No resection, Patient with 2 or more “positive” signs	4.6 mo	
He, M.-M., et al. [58]	2013	54	PG, Patient with single metastatic site	25.70 mo	0.001
		63	No surgery, Patient with single metastatic site	14.63 mo	
		39	Palliative gastrectomy, Patient with lymph node metastases	24.43 mo	0.002
		54	No surgery, Patient with lymph node metastases	9.13 mo	
		82	Palliative gastrectomy, Patient with peritoneal metastases	21.30 mo	<0.001
		81	No surgery, Patient with peritoneal metastases	10.37 mo	
		40	Palliative gastrectomy, Patient with multiple metastatic sites	15.73 mo	0.010
		121	No surgery, Patient with multiple metastatic sites	9.67 mo	
			Palliative gastrectomy	22.47 mo	*p* < 0.05 compared to no surgery, *p* > 0.05 when compared between surgery types
			Metastasectomy only	50.00 mo	
			PG + Metastasectomy	46.93 mo	
			No surgery	10.37 mo	
			PG age < 70	28.70 mo	<0.001
			No surgery age < 70	10.37 mo	
			PG age ≥ 70	23.07 mo	0.031
			No surgery age ≥ 70	10.27 mo	
Hsu, J.T., et al. [43]	2017	124	PG with chemotherapy	9.73 mo	<0.001
		83	No resection with chemotherapy	7.86 mo	
		69	PG, no chemotherapy	4.54 mo	
		57	No resection, no chemotherapy	2.93 mo	
			Age < 58 vs. ≥58		0.045
			Albumin ≤ 3 vs. >3		0.003
			LN ratio > 0.58 vs. ≤0.58		0.047
Kahlke, V., et al. [59]	2004	52	PG, Patient with major symptoms	4 mo	*p* < 0.05, when comparing pts with major symptoms to minor symptoms regardless of type of operation
		71	PG, Patient with minor symptoms	6 mo	
		12	Non-resection operation, Patient with major symptoms	5 mo	
		15	Non-resection operation, Patient with minor symptoms	5 mo	
Kim, K.H., et al. [60]	2011	42	PG + Metastasectomy	28.0 mo	*p* < 0.001 *p* = 0.024
		47	Palliative gastrectomy	15.5 mo	
		185	No surgery, chemo only	9.0 mo	
			ECOG 1–2 vs. ECOG 3–4		<0.001
			Tumor location, upper 1/3 vs. distal		0.038
Kulig, P., et al. [61]	2012	415	PG	10.6 mo	<0.001
		536	Non-resection operation	4.4 mo	
			ECOG 0–1 vs. ECOG 2–4		0.005
Kunisaki, C., et al. [44]	2008	51	PG with chemotherapy		<0.001
		95	PG		
			Differentiated vs. undifferentiated histologic cell type		0.002
			Absence vs. presence hematogenous metastases		<0.001
			No peritoneal metastases vs. CY1/P1		0.003
			No peritoneal metastases vs. P2/P3		<0.001
			Absence vs. presence of remnant lymph node metastases		<0.001
Li, J., et al. [62]	2017	46	Gastrectomy and hepatectomy	43mo	0.021
		73	Gastrectomy and RFA/TACE	37mo	
Lin, S.Z., et al. [45]	2008	183	PG	80.3% 1 yr OS	*p* < 0.001 *p* = 0.002
		112	PG with chemotherapy	85.7% 1 yr OS	
		206	Non-resection operation	33.5% 1 yr OS	
		65	PG, Patient with liver metastases	53.7% 1 yr OS	<0.001
		90	Non-resection operation, Patient with liver metastases	25.6% 1 yr OS	
		69	Chemotherapy, Patient with liver metastases	46.5% 1 yr OS	0.002
		86	No chemotherapy, Patient with liver metastases	30.2% 1 yr OS	
		114	PG Patient with peritoneal metastases	72% 1 yr OS	<0.001
		122	Non-resection operation, Patient with peritoneal metastases	14.8% 1 yr OS	
		105	Chemotherapy, Patient with peritoneal metastases	53.5% 1 yr OS	<0.001
		131	No chemotherapy, Patient with peritoneal metastases	31.2% 1 yr OS	
		33	PG, Patient with lymph node metastases	66.7% 1 yr OS	<0.001
		42	Non-resection operation, Patient with lymph node metastases	19.1% 1 yr OS	
		36	Chemo, Patient with lymph node metastases	44.4% 1 yr OS	0.450
		39	No chemo, Patient with lymph node metastases	35.1% 1 yr OS	
Lupaşcu, C., et al. [46]	2010	30	PG with chemotherapy	17.8 mo	<0.001
		25	Palliative gastrectomy	8.9 mo	
		45	Chemotherapy	6.4 mo	
Nelen, S.D., et al. [47]	2017	235	PG with chemotherapy, Patient < 70 yo	26.7% 2 yr OS	<0.001
		1106	PG Patient < 70 yo	21.6% 2 yr OS	
		1935	Chemotherapy Patient < 70 yo	6.3% 2 yr OS	
		58	PG with chemotherapy, Patient ≥ 70 yo	24.1% 2yr OS	0.027
		1415	PG, Patient ≥ 70 yo	14.7% 2 yr OS	
		640	Chemotherapy, Patient ≥ 70 yo	4.6% 2 yr OS	
		6903	Female vs. Male Patient < 70 yo		<0.001
			Anatomically isolated vs. overlapping tumor location, Patient < 70 yo		<0.001
			Well-differentiated vs. undifferentiated tumor, Patient < 70 yo		0.004
			Moderately differentiated vs. undifferentiated tumor, Patient < 70 yo		0.008
		8108	Decreasing age (continuous variable) Patients ≥ 70 yo		<0.001
			Female vs. male Patients ≥ 70 yo		<0.001
			Anatomically isolated vs. overlapping tumor location, Patients ≥ 70 yo		<0.001
			Adenocarcinoma vs. non-adenocarcinoma Patients ≥ 70 yo		<0.001
Nie, R.C., et al. [51]	2016	345	PG	11.87 mo	0.006
		402	No resection	9.27 mo	
		747	Single vs. Multi-site metastases		<0.001
			5–8 cycles vs. <5 cycles of chemotherapy		<0.001
			> 8 cycles vs. ≤8 cycles of chemotherapy		<0.001
Saidi, R.F., et al. [63]	2006	12	PG with chemotherapy	16.3 mo	0.020
		12	PG	8.5 mo	
		34	Chemotherapy	5.9 mo	
		47	No treatment	5.2 mo	
Sougioultzis, S., et al. [48]	2011	218	Palliative gastrectomy	53 mo	<0.001
		93	No resection	16 mo	
			Combination vs. single agent chemotherapy		0.013
			Absence vs. presence of liver metastases		<0.001
			Absence vs. presence of peritoneal metastases		0.024
			Grade 1–2 vs. Grade 3–4		0.007
			CA 72-4 ≤ 7 vs. >7		0.034
			Not elevated vs. elevated LDH		<0.001
			Weight loss ≤ 5% vs. >5%		<0.001
			No blood transfusion vs. blood transfusion		0.001
		66	Low risk (0–2 factors from MV analysis)	76 mo	<0.001
		197	Intermediate risk (3–6 factors)	40 mo	
		48	High risk (7–9 factors)	11 mo	
Tiberio, G.A.M., et al. [50]	2015	98	Palliative gastrectomy	6.6 mo	0.009
		44	Non-resection operation	3 mo	
			T1–2 vs. T3–4		0.036
			1 hepatic metastasis vs. 2–3 hepatic metastases		0.003
			Chemotherapy vs. no chemotherapy		<0.001
Tokunaga, M., et al. [38]	2012	82	PG	13.1 mo	0.410
		66	No resection	12.0 mo	
		121	Chemotherapy	13.7 mo	0.048
		27	No chemotherapy	7.1 mo	
			ECOG 0–1 vs. ECOG 2–4		<0.001
			Macroscopic type 3 vs. others		0.006
		18	P1 metastasis with R0 resection	26.4 mo	<0.001
		16	P1 metastasis with R1/R2 resection	12.3 mo	
		6	P1 metastasis with chemotherapy only	12.5 mo	
Yang, K., et al. [64]	2015	114	PG, all patients	14.0 mo	<0.0001
		153	Non-resection, all patients	8.57 mo	
			PG with chemotherapy	18.37 mo	<0.0001
			Non-resection with chemotherapy	11.77 mo	
			PG without chemotherapy	8.90 mo	<0.0001
			NR without chemotherapy	4.73 mo	
			P1–2 vs. P3 peritoneal metastases		<0.0001
Yao, G.L., et al. [65]	2015	18	PG + hepatectomy (H1 or H2) + HAC	24 mo	0.002
		31	PG + HAC	12 mo	
Yuan, S.Q., et al. [49]	2017	30	Palliative gastrectomy	23.6 mo	0.034
		30	No resection	13.8 mo	
			First line chemotherapy, ≤5 cycles vs. >5 cycles		<0.001
		20	R0 resection	43.6 mo	<0.001
		10	R1/2 resection	11.27 mo	
		30	No resection	13.8 mo	
Zhang, J.Z., et al. [66]	2011	197	PG	16.4 mo	<0.05
		78	Bypass	5.7 mo	
		102	Exploratory laparotomy	4.7 mo	
		152	No surgery	5.5 mo	
		114	PG + D0 lymphadenectomy	16.1 mo	>0.05
		83	PG + D2 lymphadenectomy	16.8 mo	

^†^ Positive (+) sign defined as T+ unresectable tumor, P+ peritoneal mets, N4+ lymph node mets, H+ hepatic mets. mo = Months, yr = Years.

**Table 3:** Comparison of operative vs. non-operative interventions to control symptoms in late-stage GC *.

Intervention	30 Day Mortality (%)	Morbidity/Complications (%)	Return to Oral Intake (Days)	Length of Stay (Days)	Clinical Success (%)
PG	0–24.1	10.4–88.9		11.56–41.0	
SB	0–6.0	22.0–80.0	2.9–7.0	7.0–19.0	
ES	0	16.–45.9			81.1–97.0
PG vs. SB	0–18.0 2.5–19.2	19.0–42.1 2.5–21.2		10.7–31.5 7.0–12.5	
SB vs. ES		10.0–30.0 4.5–84.8	4.9–8.0 1.0–2.1	7.7–20.6 0.94–12.8	77.3–98.6 77.3–100

* Data not provided uniformly across all studies. Values represent ranges based on available reported outcomes. PG = Palliative Gastrectomy, SB = Surgical Bypass, ES = Endoscopic Stenting.

## Data Availability

No new data were generated. All data across compiled studies were synthesized in our figures and conclusions.
